# Cone Beam Computed Topographic Evaluation and Endodontic Management of a Rare Mandibular First Molar with Four Distal Canals

**DOI:** 10.1155/2014/306943

**Published:** 2014-12-01

**Authors:** Nidhi Sinha, Bijay Singh, Akshay Langaliya, Nitin Mirdha, Irfanul Huda, Ashwin Jain

**Affiliations:** ^1^Department of Conservative Dentistry and Endodontics, Jodhpur Dental College General Hospital, House No. 253, Ashapurna Enclave, Pal Bypass, Near DPS School, Jodhpur, Rajasthan 342001, India; ^2^Department of Prosthodontics, Jodhpur Dental College General Hospital, Jodhpur, Rajasthan 342001, India; ^3^Department of Conservative Dentistry and Endodontics, AMC Dental College, Ahmedabad, Gujarat 380008, India; ^4^Department of Prosthodontics, Sarjug Dental College, Darbhanga, Bihar 846003, India; ^5^Department of Conservative Dentistry and Endodontics, YMT Dental College and Hospital, Navi Mumbai, Maharashtra 410210, India

## Abstract

Root canal system is complex to understand because of its unpredictable nature. It differs for different teeth and for the same teeth in different individuals. Successful endodontic therapy thus depends on the clinician's ability to anticipate and look for these variations. A mandibular first molar with six root canals represents a rare anatomical variant, particularly when four canals are found in distal root. This case report discusses successful nonsurgical endodontic management of two-root mandibular first molar with four distal canals and two mesial canals reported for the first time in Indian population. Cone beam computed tomography was used as a diagnostic method to confirm the position and presence of 4 root canals in the distal root.

## 1. Introduction

Blayney in 1932 very aptly said that “probably the first requisite for successful operation is that the operator should be thoroughly familiar with the anatomy of the region” [1]. The same holds true for any tooth requiring endodontic treatment. Success rate of endodontically treated teeth has been reported to be 87.79% and it is still lower for mandibular first molars (MFM) reaching around 81.48% [2]. Incidences of missed roots or canals among the teeth requiring retreatment have been reported to be as high as 42% [3]. In case of MFM 86% of missed canals are found in the distal root and 14% in the mesial roots [4]. This could be attributed to the considerable anatomic variation and abnormalities regarding number of roots and root canals, intracanal communications, and curvatures not visible in conventional radiographs [5].

Complete disinfection of the entire coronoradicular space harbouring microorganisms and infected or necrotic pulp is essential for successful endodontic treatment. This necessitates clinicians to have a thorough working knowledge of radicular tooth anatomy along with a three-dimensional appreciation of internal anatomy of root canals with its possible variations [6]. Advanced diagnostic tools like cone beam computed tomography (CBCT) can facilitate the confirmatory diagnosis of root canal morphologic aberrations thereby helping clinicians to accurately locate, explore, and disinfect root canal system [7].

The most common root canal morphology of MFM is the presence of 2 roots with either 3 or 4 root canals [8]. There are many reported cases and studies in literature regarding the presence of three or four canals in the mesial roots of MFM but limited data is available for more than 2 root canals in distal root [8, 9]. The incidence of a third canal in distal root itself is a rare occurrence with a prevalence rate of 0.2% to 3% in different ethnic groups [10]. Furthermore literature regarding four canals in distal root is absolutely scarce with only a few reported cases till date [11, 12]. This case report aims to report a rare anatomical variation in the root canal system of MFM with four root canals in the distal root confirmed by CBCT.

## 2. Case Report

A female patient aged 18-year reported to the Department of Conservative Dentistry and Endodontics of Jodhpur Dental College General Hospital, Rajasthan, India, with the chief complaint of severe pain and swelling in the lower right back tooth region since 2 days. She also complained of food lodgement in the same tooth since 6–8 months. Patient's medical history was noncontributory and vital signs were within normal limit.

Extra-oral examination showed a soft diffuse swelling on the right lower border of the mandible extending up to the angle of the mandible. Clinical examination revealed a deep proximoocclusal carious lesion in relation to right MFM. The tooth was severely tender on vertical percussion and palpation. There was no sinus tract present in the involved tooth. The periodontal status was normal (probing depth < 3 mm) with no mobility. The tooth gave a negative response to all three pulp vitality tests (electric, cold, and heat).

**Figure 1:**
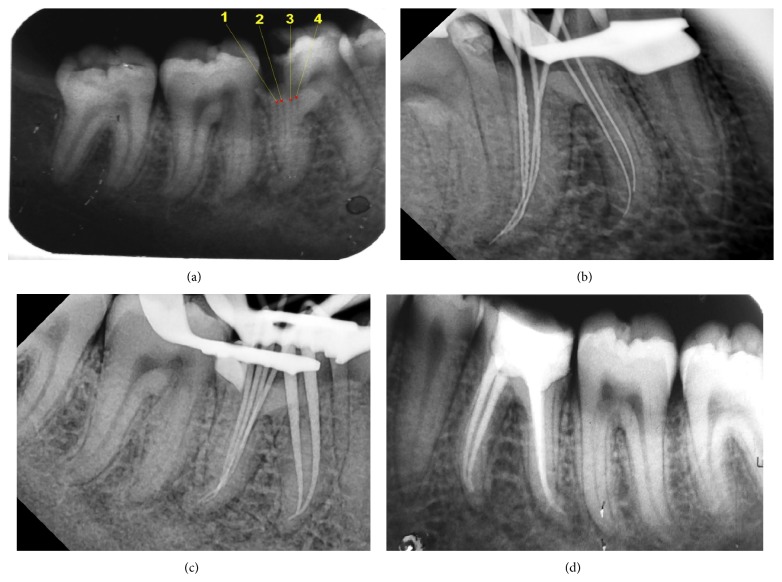
(a) Preoperative intraoral periapical radiograph 1-distobuccal I, 2-distobuccal II, 3-middistal, and 4-distolingual. (b) Radiograph showing working length determination of six root canals. (c) Master cone radiograph. (d) Postobturation radiograph.

Radiographic examination (Figure 1(a)) revealed a deep carious lesion involving the distal pulp horn and widening of the periodontal ligament space. Intraoral periapical radiograph was indicative of four canals in the distal and two in the mesial root. Clinical and radiographic findings lead to a diagnosis of acute exacerbation of chronic periapical abscess and treatment planned was of nonsurgical endodontic treatment. Inferior alveolar nerve block was administered to the patient with 2% Lignocaine with 1 : 100,000 epinephrine (Lignox 2%, Indoco Remedies Ltd., Mumbai). After isolation with rubber dam (Hygenic Dental Dam, Colténe Whaledent, Germany), access was gained into the pulp chamber with a nonend cutting tapered fissure bur (Endo-Z, Dentsply Maillefer, Ballaigues, Switzerland). Caries was excavated and the pulp chamber was flushed with 2.5% sodium hypochlorite to remove the debris. Access cavity was extended more buccolingually to improve the visibility of the extra canals giving it a rectangular shape. Partial coronal pulp chamber calcification was encountered on entering the pulp chamber. Initially the dentinal map was explored using a DG 16 endodontic explorer (Hu-Friedy, Chicago, IL) which revealed 4 canal orifices mesiobuccal, mesiolingual, distobuccal, and distolingual. Dentinal bridge connecting the two distobuccal, middistal, and distolingual orifices was removed using ultrasonic tips (Proultra tips, Dentsply, and Tulsa Dental). Finally four canals were located in the distal root-distobuccal I, distobuccal II, mid-distal and distolingual. Initial canal-negotiation was carried out with numbers 6, 8, and 10 K-files (Mani, Inc., Tochigi, Japan). Working length was estimated with an apex locator (Root ZX mini-J Morita MFG. Corp., Kyoto, Japan) and confirmed with digital radiograph (Figure 1(b)). Cleaning and shaping were performed with rotary NiTi files (Twisted files, SybronEndo, Orange, CA, USA) and RC prep (Premier Dental Products, Norristown, PA, USA; 15% EDTA and 10% urea peroxide in a base of carbowax) as a lubricant. Mesial root canals (mesiobuccal and mesiolingual) were prepared to a master apical file size of 35 with 6% taper and distal canals (distobuccal I, distobuccal II, middistal, distolingual) to a master apical file size of 25 with 4% taper. Irrigation was performed with 5.25% sodium hypochlorite and 2% chlorhexidine (Sigma Chemicals, St. Louis, MO, USA) and saline was used alternatively in between them. Side-vented irrigation needles [R C Twents irrigation needle, Prime Dental Products Pvt. Ltd., Mulund, Mumbai] were used during the irrigation. Care was taken to keep the needle tip of the radiographic apex 1 mm short. 2% chlorhexidine gel was placed as the interappointment dressing and the tooth was temporized with Cavit-G (3M ESPE, United States).

**Figure 2:**
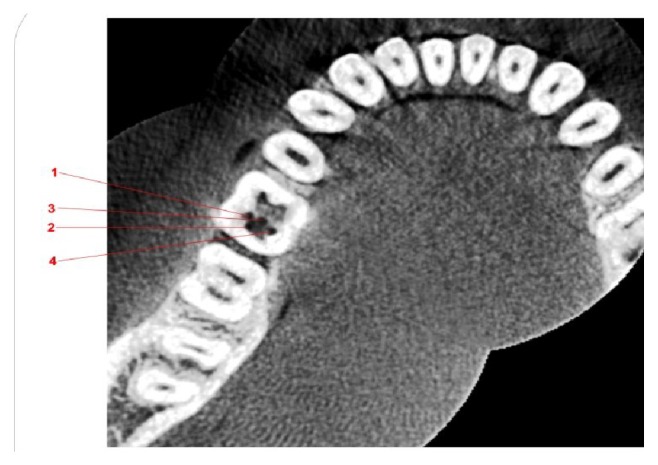
Enlarged CBCT axial section at the coronal one-third showing four distal and two mesial root canals, 1-distobuccal I, 2-distobuccal II, 3-middistal, and 4-distolingual.

CBCT was advised to further substantiate this rare root canal anatomy and for the better understanding of the root canal configuration. An informed consent was obtained from the patient before proceeding with the CBCT of the mandible. The examination was performed with a CBCT Unit Kodak 9300 3D Imaging system with a voxel size of 0.20 mm × 0.20 mm × 0.20 mm and 5.5 cm field of view. The images were reconstructed using C S 3D Imaging Software v 3.2.9 (Carestream Dental, US). Complete morphology of the root canal system was obtained in coronal, axial, and sagittal sections of 0.2 mm thickness. CBCT (Figure 2) revealed 4 canal orifices which merged into two canals apically in the distal root and two separate canals in the mesial roots.

**Figure 3:**
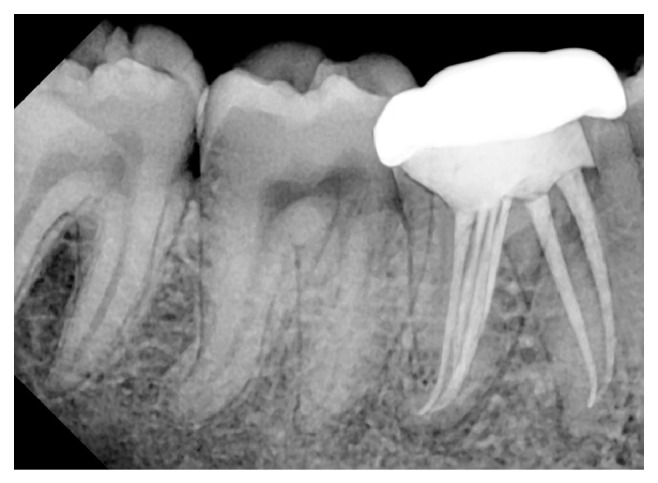
15-month follow-up radiograph.

Next appointment was scheduled after 7 days, during which patient was asymptomatic. Following anaesthesia and isolation intracanal medicament was removed with copious irrigation and ultrasonic files. The canals were flushed with 10 mL of 5.25% hypochlorite and activated with Endoactivator (Dentsply, Maillefer, Ballaigues, Switzerland) for 2-3 minutes in each canal. Final irrigation was done with 17% ethylenediaminetetraacetic acid (EDTA) solution and dried with absorbent paper points (Dentsply, Maillefer, Ballaigues). Master cone radiograph (Figure 1(c)) was taken followed by obturation by lateral condensation technique using* Gutta percha* (Dentsply, Maillefer, Ballaigues) and AH plus sealer (Dentsply, DeTrey, Germany). Owing to the close proximity the distal canals to each other, they were not separately appreciable in the obturation radiograph but they could be clearly visualized in the CBCT and preoperative radiograph. Core buildup was done with composite (Para-core, Coltene, Whaledent Inc, USA) and the patient was advised a full coverage crown (Figure 1(d)). Patients were asymptomatic and the tooth was in function at 15-month follow-up (Figure 3). Patient is under active follow-up thereafter.

## 3. Discussion

Literature has innumerable studies reporting consistently teeth with aberrant root canal morphologies [6–12]. Internal anatomy shows great variation not only in the number of roots and their shape but also in the number, position, and location of root canals. Generally dentists with a predetermined approach towards treating root canals are unable to foresee these variations leading to unpleasant complications and unfavourable treatment outcomes. Etiology of endodontic failure is multifactorial but majority of them can be attributed to the persistence of microorganisms and inability to access the full anatomy of root canal system. Missed canals remain unfilled even after treatment and thus act as nidus for infection containing pulp tissue remnants, microbiota, and irritants which inevitably compromise treatment outcome [13].

Martínez-Berná and Badanelli reported the first case of a third canal in the distal root of MFM and termed it as distocentral root canal [14]. Its prevalence based on racial distribution has been reported to be 1.7% in Indian and Turkish population; 0.2% in Senegalese; 0.3% in Jordanian; 0.7% in Burmese; 1.6% in Thai; and 3% in Sudanese population [10]. A spiral CT study of 125 MFM in an Indian population concluded that none of the teeth showed the presence of three distal canals [15]. MFM with 4 distal canals has only been reported twice first by Ghoddusi et al. in 2007 and recently by Baziar et al. in 2014. Both patients were of Iranian origin [12, 13]. This is the first reported case of such a variation in a patient of Indian origin.

Variation in the distal root of MFM has been reported to be higher in younger age group (less than 31 years) which is in accordance with the present case report. Pattanshetti et al. concluded that the frequency of finding second canal in the distal root of MFM reduces as the age advances. He presumed that the tooth exposure to insults like caries, attrition, and erosion with age leads to calcification of the orifice or canal itself thus making it difficult to locate the canals [10, 16]. Contrary to this, Baziar et al. [12] in 2014 reported a case of 4 root canals in distal root in an older patient (43 years). Furthermore, in terms of sidewise prevalence, a much higher prevalence has been reported in the right side [17]. The same was observed in the present case report.

Early eruption of MFM makes it the most frequent tooth requiring endodontic or restorative procedures. Presence of iatrogenic and pathological problems and heavy occlusal forces lead to frequent pulp chamber calcification associated with it [18]. Similar calcification was seen in the present case which required the use of ultrasonics (US). US aided not only in the removal of the calcification in the orifices but also in the exploration and negotiation of the root canals. US were used along with hypochlorite which has a collagen dissolving action, whereas US themselves have a physical action of dislodging the calcifications. The use of US in unearthing such canals has been reported in previous studies [19].

MFM was reported to have a canal configuration 1-1 (62.7%), followed by 2-1 (14.5%) and 2-2 (12.4%). Furthermore 77.2% of the distal roots had one foramen while 22.2% had two foramina [20]. 74.8% of distal roots have been reported to be flattened mesiodistally with a much complex ribbon shaped distal root configuration. Clinical applicability of this observation is that access modification to a more rectangular or trapezoid form shifting to the mesiobuccal quadrant may be required when extra canals are expected [13].

The third canal if present is frequently located centrally between the buccal and lingual roots with a smaller diameter compared to the other two canals [15]. The maximum diameter size of apical foramen in case of a single canal or two separate canals is 0.31 mm and 0.25 mm, respectively. Clinical implication of this finding suggests that root canal configuration with multiple number of canals should be prepared more conservatively as they have smaller apical diameter. Cleaning and shaping should also be correlated with the microbial status of the tooth implying that infected canals may require larger apical preparation for efficient irrigation and disinfection [21].

Various methods advocated for additional canal location include knowledge of law of symmetry and canal location, use of endodontic explorers like DG16 and microopener, Champagne test, fibre-optic illumination dental endoscopy and orascopy, use of magnification, visualisation endograph using Ruddle's solution, magnetic resonance microscopy [20]. Use of Stropko irrigator fitted with a 27-Gauge notched irrigating needle with sequential application of 17% ethylenediaminetetraacetic acid (EDTA) and 95% ethanol needle for effective cleaning and drying of pulp chamber floor for better visualisation has been advocated by Stropko [22].

Conventional radiography is of limited significance in cases with complex root anatomy as it produces images in only two dimensions, usually in the mesiodistal direction. They can aid in the identification of these aberrancies but are of not much significance for the exact location of the canals [23]. An ex vivo investigation using digital radiography concluded that endodontists failed to locate at least one canal in 40% of teeth [24]. Tu et al. reported a much higher detection of extra roots in the MFM by CBCT in comparison to conventional radiography [7]. CBCT is a unique noninvasive technique which provides undistorted three-dimensional information of the root canal anatomy, at a reduced dose and cost in comparison to multidetector CT [9]. In addition it permits the clinician to look at multiple sections of the roots and their canals at different levels and angulations [23].

The present case reports four root canal orifices in the distal root of MFM with two apical terminations which could be classified as type XIV canal configuration according to Sert and Bayirli [25]. This canal configuration is similar to only one previously reported case [12]. Vertucci found the proximity of the canal orifices to each other as an indicative factor of whether they join or remain as separate canals. It was found that when the separation was more than 3 mm the canals remained separate whereas they joined together if it was lesser. [8]. When the distance between root canal orifices reduces they were found to join more coronally. This finding correlated with our observation where the two distobuccal, the middistal, and distolingual canals fused in the coronal third itself. The fact that observation was not evident clinically and radiographically, but was evident with a CBCT, emphasizes the vital role of this diagnostic tool.

## 4. Conclusion

Excellence in endodontics can be achieved by comprehensively understanding the key elements essential for successful treatment and by critically evaluating the etiology of failures. Endodontic treatment of multirooted teeth is always challenging to the clinicians due to the enormous variations associated with them. Inability to locate and access aberrant root canals results in failures. Clinician with good biological rationale, thorough knowledge of dental anatomy, careful interpretation, and utilisation of diagnostic tools integrated with skill and experience can deliver successful treatment to the patient. The use of US and CBCT in the above case illustrates that endodontic research and technology are continually evolving and to provide best results to our patients it is our duty as clinicians to be a part of this evolution and to apply and utilize these technological advancements to efficiently identify, disinfect, and obturate root canal.
